# Aspects associated with waterpipe smoking in Iranian youths: a qualitative study

**DOI:** 10.1186/s12889-021-11675-y

**Published:** 2021-09-07

**Authors:** Hamid Jafaralilou, Arman Latifi, Mehdi Khezeli, Atefeh Afshari, Farahnaz Zare

**Affiliations:** 1 Department of Public Health, Khoy University of Medical Sciences, Khoy, Iran; 2 Department of Public Health, Maragheh University of Medical Sciences, Maragheh, Iran; 3 Social Development and Health Promotion Research Center, Health Institute, Kermanshah University of Medical Sciences, Kermanshah, Iran; 4 Department Of Community Health Nursing, School of Nursing And Midwifery, Nursing Midwifery Care Research Center, Isfahan University of Medical Sciences, Isfahan, Iran; 5 Clinical Research Development Center, Imam Khomeini and Mohammad Kermanshahi and Farabi Hospitals, Kermanshah University of Medical Sciences, Kermanshah, Iran

**Keywords:** Beliefs, Qualitative research, Perception, Self-efficacy, Water pipe smoking

## Abstract

**Background:**

Waterpipe is one of the oldest methods of tobacco smoking, which has become the public health challenge, especially in the Eastern Mediterranean countries such as Iran. This study aimed to investigate the waterpipe smoking (WPS) in the young people of Kermanshah in 2020, using a qualitative method.

**Methods:**

This was a qualitative study conducted with the approach of content analysis. Participants were young waterpipe user aged 17 to 25 years selected by purposeful sampling method in Kermanshah city, located in the west of Iran. Data were collected through semi-structured interviews in face-to-face and audio-recorded methods based on an interview guideline during June to August 2020. Then researchers transcribed verbatim and analyzed the content of the interviews thematically.

**Results:**

In this study, 23 young people who were waterpipe users at the time of the study participated. The results showed that social aspects in three sub-categories were involved in WPS including “socio-cultural aspects”, “socio-environmental aspects”, and “social relations”. Individual aspects of waterpipe use as second category also consisted of two sub-categories including “motivational aspects” and “lack of psycho-protective aspects”.

**Conclusions:**

It seems that the implementation of the policy of reducing access to waterpipe in public environments is effective in reducing waterpipe consumption. It is suggested that educational and interventions, based on targeted models and theories be implemented in order to increase young people’s belief and perception on dangers of WPS, and to improve their self-efficacy to smoking cessation.

## Introduction

Smoking is a major risk factor for noncommunicable diseases (NCDs) such as cancer and heart diseases, causing more than 7 million deaths worldwide each year [1, 2]. Waterpipe known as Ghalian in Iran, is one of the oldest methods of tobacco smoking, which has now become one of the major public health challenges in the world, especially in the Eastern Mediterranean countries, including the Arab countries, Turkey, and Iran [3, 4]. Since the late 1990s, waterpipe has been introduced as an inexpensive and social method of smoking, especially among young people and students [5]. It is estimated that the prevalence of waterpipe smoking (WPS) in youths Eastern Mediterranean region (EMR), about 15% of use waterpipe [6]. A longitudinal study among young people in EMR showed a 40% in prevalence of WPS within 2 years of follow-up [7]. In Iran, various studies have reported high rates of WPS among young people, especially college students. For example, in studies by Latifi et al. in [8], Karimi-Afshar et al. [9], and Ghafouri et al. [10], more than a third of youths had a history of WPS or were current waterpipe users.

In Iran, similar to Arab countries, waterpipe has less social stigma than cigarettes. Although, in the past, waterpipe was more common among the elderly, today it has become very popular among young people as a means of leisure, social gathering and communicating [11, 12]. Studies have shown that waterpipe users believe that it is healthier than cigarettes, is not addictive, and its smoke is more enjoyable than cigarettes due to the use of Moassel or fruit-flavored tobaccos [13, 14]. However, studies have shown that WPS is associated with a number of harmful health consequences such as lung and esophageal cancers, respiratory diseases, low birth weight, and oral diseases [15]. In addition to chronic diseases, waterpipe exposes people to infectious diseases such as tuberculosis, and viral infections including hepatitis and herpes, as a result of sharing between waterpipe users [5, 12, 16]. Despite the health risks of waterpipe, its use is increasing and has reached alarming levels in some countries [15, 17].

This study was conducted in the Kermanshah city in western Iran, where the majority of residents are Kurds. Few studies have been conducted on the prevalence or desire of WPS in Kurdish people, but the prevalence of hookah use, especially at a young age, is significant and alarming in few studies. Bashirian et al., in a study showed that 20.4% of female adolescents in Kermanshah were current WP users [18]. Another study in Kermanshah found that 36.1% of high school boys reported ever hookah use and 17.1% mentioned WPS in the past month [19].

The development of effective intervention strategies to restrict the increasing use of waterpipe requires a clear understanding of the factors influencing this behavior [20]. It should also be noted that WPS depends on culture, ethnicity, and other social environments [21, 22]. Qualitative studies are the most important tools for understanding culture-based topics that can assess the WPS in a particular geographical or cultural area and provide rich information about the related factors [23]. Therefore, we conducted this study to investigate the WPS in the young people of Kermanshah in 2020 using a qualitative method.

## Materials and methods

### Design

This was a qualitative study conducted with the approach of content analysis to explore the participants’ perspectives on the nature, and aspects associated to the WPS.

### Participants

Participants of this study were young people aged 17 to 25 years in Kermanshah city, located in the west of Iran. They were Muslim Kurds or Persian and were also current waterpipe users. We used purposeful sampling method to select the participants by which the eligible waterpipe users were invited to interview in coffee shops or traditional restaurants. Criteria for entering the research being 17 to 25 years old; both men and women, being current WP smoker i.e. WPS at least once in the last 30 days [24], having the ability to speak to record the interview, giving informed consent to participate in the study. The study did not consider any limitations on factors such as financial status, family status of WPS, marital status, level of education and some other underlying factors, although they may have been confounder factors.

### Data collection

The data collection method was semi-structured interview based on an interview guideline during June to August 2020. Interview questions were designed to gather rich descriptions of experiences, and to achieve research objectives. It consisted of 10 questions originally, which sometimes increased according to the interviewees’ responses (please refer to Table 1). Adequate sample size in qualitative studies is a subject-oriented issue based on information needs. In the present study, data collection continued until information saturation, which occurred with 23 interview. The male and female interviewers were members of research team and health promotion specialist.

**Table 1:** Questions asked in the interview

Number	Question content
1	Do you think the waterpipe smoking has social aspects? Can you explain?
2	What social aspects of waterpipe smoking apply to you? Please explain.
3	Do you think the waterpipe smoking has individual aspects? Can you explain?
4	What individual aspects of waterpipe smoking apply to you? Please explain
5	What increase your desire to waterpipe smoking?
6	What reduce your desire to waterpipe smoking?
7	What subject (s) are involved in starting your own waterpipe smoking?
8	Do you prefer to smoke waterpipe at home or in public spaces? Why?
9	Do you think waterpipe is more harmful or cigarettes?
10	Have you ever tried to quit waterpipe? What was the result?

It should be noted that the content of the interview guideline was confirmed by experts in related fields (three health education specialists, two psychologists, a sociologist, and a health promotion specialist).

### Procedures and ethics

The interviews were conducted face-to-face and lasted 30 to 45 min in coffee shops or traditional restaurants in Kermanshah city. The interviews were recorded after obtaining written informed consent from the participants. All interviews were conducted in the language of the interviewee (Kurdish or Persian) for mutual and deeper understanding of the subject under study. Non-verbal messages of individuals and their body language were also recorded by memoing. We asked the interviewees to ask any questions they had at the time of the interview. Recording the interviews were also paused if interviewees wanted to make a comment out of the record. This study received ethics approval from the Research Ethics Committee of Kermanshah University of Medical Sciences (KUMS.REC.1396.751). We confirm that all methods related to the human participants were performed in accordance with the Declaration of Helsinki and approved by Research Ethics Committee of Kermanshah University of Medical Sciences.

### Data analysis

In the present study, according to the inductive approach, we transcribed verbatim the content of the interviews immediately after each interview considering the nonverbal clues such as Anger, sadness, smiles, regrets, etc. Three members of the research team read the texts separately, and in the first step, called the initial encounter, notes, associations, summaries, labels, and questions were noted in the left margin of the pages. In the second stage, which was the stage of identifying and labeling sub-categories, meaning units were determined and labeled. In the third stage, sub-categories were organized and based on conceptual similarity, the main categories were finally determined.

### Trustworthiness

In the present study, Lincoln and Guba’s Evaluative Criteria was used to assess the trustworthiness of the data including credibility, transferability, confirmability, and dependability [25]. The two methods used to assess credibility were member checks and peer debriefing. In the member checks, a summary of the interview was returned to the participants to review the agreement. In cases where there was disagreement between the researchers and the participant, research team attempted to reach a common understanding through interaction with subjects. In peer debriefing, we invited experts in the fields of health, psychology, and sociology to comment on the results of the study and the categories extracted. To meet the transferability criteria, we used thick description of subject, and purposive sampling with maximum variance in term of education, job, gender, marital status, and age. Dependability was met by audit trails in the implementation of the research protocol including sample selection, data collection and analysis, and matching the findings. Peer check as a confirmability method was used to assess the confidence that the results would be confirmed by other researchers.

## Results

In this study, 23 young people participated who were waterpipe users at the time of the study. The mean (SD) age of participants was 22.08 (2.74) years. The majority of participants were male (16; 69.56%), single (17; 78.26), had university education (12; 52.17%), and unemployed (13; 56.52%). Details of demographic characteristics of the interviewees is provided in Table 2.

**Table 2:** Demographic characteristics of the interviewees (*n* = 23)

Code	Age	Gender	Marital status	Education	Employment
1	23	Male	Single	University	Unemployed
2	20	Male	Single	Under diploma	Unemployed
3	19	Female	Single	University	Unemployed
4	18	Male	Single	Under diploma	Unemployed
5	25	Male	Married	University	Employee
6	23	Male	Married	Diploma	Self-employed
7	20	Male	Single	University	Unemployed
8	19	Male	Single	Under diploma	Unemployed
9	22	Female	Single	University	Unemployed
10	25	Female	Married	University	Housewife
11	25	Male	Married	University	Employee
12	24	Male	Single	University	Unemployed
13	23	Male	Single	University	Self-employed
14	26	Male	Single	University	Unemployed
15	21	Female	Single	Diploma	Unemployed
16	23	Male	Single	Diploma	Self-employed
17	21	Female	Married	Diploma	Housewife
18	21	Female	Single	University	Self-employed
19	25	Female	Single	Diploma	Unemployed
20	22	Male	Single	Diploma	Self-employed
21	25	Male	Married	University	Self-employed
22	20	Male	Single	Under diploma	Unemployed
23	18	Male	Single	Diploma	Unemployed

Interviews with 23 young waterpipe users resulted in information saturation, and data replication. The initial codes were extracted according to the results of the interviews, and subsequently sub-categories and main categories were identified. In this study, two main categories were “social aspects of waterpipe use”, and “individual aspects of waterpipe use” along with five sub-categories. Each of these categories had one or more subcategories and the semantic codes, presented in Table 3.

**Table 3:** Categories, sub-categories and codes related to waterpipe use in the subjects

Categories	Sub- Categories	Codes
**Social aspects of waterpipe use**	Socio-cultural aspects	*Social norm* *Family norm*
	Socio-environmental aspects	*Accessibility* *Unemployment* *Leisure time*
	Social relations	*Peer influence*
**Individual aspects of waterpipe use**	Motivational aspects	*Appeal* *Relaxing*
	Lack of psycho-protective aspects	*Risk perception* *Self-efficacy*

### Social aspects of waterpipe use

In this category, three aspects or sub-categories including “socio-cultural aspects”, “socio-environmental aspects”, and “social relations” were extracted, each contained one or more semantic codes.

#### I. Socio-cultural aspects

The analysis of the interviews resulted in two aspects related to culture in the subjects, which were: “social norm” and “family norm”.

##### Social norm

Fourteen participants believed that waterpipe is a popular social phenomenon in the community in which they lived and among their generation. Some people believed that waterpipe is not a stigma like cigarettes and there was no need to hide the WPS from others in society.

*Participant # 22, single, male: “I smoke waterpipe like everyone else. I don’t think it’s wrong. Waterpipe is so popular”.*

##### Family norm

According to nine participants, WPS in families has become a common issue and in five cases was reported as a family accepted behavior. They believed that the culture of WPS is ingrained in families and there is no prohibition on the use of waterpipe by parents, children, or spouses.

*Participant # 3, single, female: “ Participant # 3: “I smoke waterpipe at home, with my father and grandfather. My father says it’s better for me to smoke waterpipe at home than outside”.*

#### II. Socio-environmental aspects

In this study, we categorized meaning codes related to economic aspects, physical environment, access, and other issues related to WPS in the environment in this sub-category. Specifically, three semantic codes including accessibility, unemployment, and leisure time were included in this sub-theme, which are mentioned below:

##### Accessibility

In this study, 19 participants reported easy access to waterpipe in multiple places such as coffee shops and restaurants as one of the reasons for their WPS.

*Participant # 4, single, male: “We do not need to look for a coffee house. Now, in addition to the Taq-e Bostan [a historical-recreational place in the north of Kermanshah city with a lot of coffee shops], there are many cafeterias in every area and neighborhood of the city. The prices may be different, but waterpipes can be found everywhere in the city”.*

##### Unemployment

One of the issues related to WPS raised by participants was the lack of an activity as a “job” or in other words “being unemployed”. According to many young people, unemployment has affected both their inclination towards waterpipe and the rate of WPS.

*Participant # 1, single, male: “I’m unemployed and have no a job. In fact, I started smoking waterpipe due to the unemployment. If I had a job, I would not be here at this time and was at workplace.*

##### Leisure time

Lack of facilities and recreational places to spend leisure time was one of the environmental problems in the city of Kermanshah, which was mentioned by nine participants as a factor in their tendency towards WPS.

*Participant # 12, single, male: “There are not many entertainment places in this city to go there. We are young and need fun. “Waterpipe and coffee houses have become our entertainment, work and life.”*

#### III. Social relations

In this study, social relations played a pivotal role in the initiation or continuation of WPS, and according to the interviews, the semantic unit had a prominent role in the influence of peers.

##### Peer influence

We found that influence of peers or friends was one of the most important aspects related to WPS in young people. Twenty of the interviewees stated that they smoked waterpipe for the first time with the compliments or insistence of their friends, and now they go to the cafeterias with their friends to use waterpipe. Some of them mentioned that the most important motivation for smoking waterpipe was to be with friends and to be among them.

*Participant # 18, single, female: “I first came to the coffee shop with my classmates. I really did not intend to smoke a waterpipe. Girls and boys all smoked there, and I was tempted to smoke a waterpipe. Then it continued. It was not a bad experience”.*

### Individual aspects of waterpipe use

In this study, the content analysis of the interviews showed that in addition to social issues, some individual aspects also influenced the tendency of people to WPS. This category contained two sub-categories including “motivational aspects” and “lack of psycho-protective aspects”; each contained two semantic codes.

#### I. Motivational aspects

Content analysis of the interviews revealed items that pointed to the attractiveness of WPS, including the smell and taste of fruit tobacco playing with smoke and creating smoke ring, and the calmness due to WPS. According to the semantic similarities, we put these aspects in two meaning codes including Appeal and Relaxing.

##### Appeal

From the point of view of all participants, waterpipe was an attractive means of smoking. The most important antisocial factor in the attractiveness of waterpipe was the taste and smell of waterpipe tobacco, and the resulting smoke.

*Participant # 17, married, female “The taste of waterpipe is very important to me. In fact, I love the waterpipe because of its taste and smell!”*

##### Relaxing

Twelve of the interviewees believed that smoking a waterpipe makes them feel calm. Some believed that waterpipe nicotine is far less than cigarettes, but it is far more sedative. To justify this, eight of them believed that they do not take waterpipe smoke into their lungs and only enjoy smoking it, and that enjoying it makes them feel calm.

*Participant # 23, single, female: “When I have a problem with my family, I smoke a waterpipe to relax and temporarily I forget my problems. It is better to say that I try to forget.*

*Participant # 5, married, male: “No one can say that waterpipe is not relaxing. Maybe because of nicotine, I relax when I smoke waterpipe. By the way, when I do not smoke, I feel upsetting.”*

#### II. Lack of psycho-protective aspects

We found that the participants attributed the lack of some psychological factors to their tendency to use waterpipe or the rate of its use. Two semantic codes including risk perception and self-efficacy were classified in this sub-theme.

##### Risk perception

We categorized some perspectives such as low perceived risk of waterpipe, comparison of side effects of cigarette smoking with waterpipe, reducing health effects of waterpipe by mechanism of passing smoke through water, and low nicotine of waterpipe in this category.

*Participant # 13, single, male: “Everyone knows that waterpipe is much better and less harmful than cigarettes. I did not smoke cigarettes but I know cigarettes are very harmful. I do not bother much when I smoke waterpipe.”*

*Participant # 12, single, male: “Do you know why waterpipe is less harmful? Because its smoke passes through the water and its poisons and harmful substances are removed. Light waterpipes such as lemon-mint or chewing gum are not harmful”.*

##### Self-efficacy

One of the important issues introduced by 11 of the participants was their lack of resistance to WPS and their low motivation to quit waterpipe. Some of them reported that although they were aware of some harms of waterpipe, their inability to resist the temptation of waterpipe led them to start using waterpipe. We named these aspects as low self-efficacy.

*Participant # 21, married, male: “Although I smoke waterpipe for fun, but I cannot quit. Every time I want to quit waterpipe, two or three days later I am tempted again.”*

*Participant # 16, single, male: “I do not think about giving up waterpipe because I know I cannot quit. When I like it, it is difficult for me to quit. I see no reason to quit.”*

## Discussion

The present study was a content analysis study that was conducted to understand the WPS and related aspects among young people in Kermanshah. The results of this study showed that social aspects in three sub-categories were involved in WPS include: socio-cultural aspects, socio-environmental aspects, and social relations. Individual aspects that referred to waterpipe tool, or psychological aspects were also categorized in another category, including two sub-categories: motivational aspects and lack of psycho-protective aspects.

### Social aspects of waterpipe use

Various studies have shown that social issues are among the most important aspects of WPS, which include issues such as social popularity, the impact of family and peers, and access to waterpipe [21, 26]. Other studies have shown that in Middle Eastern culture, WPS is recognized as a cost-effective and pleasant social activity with low health risks [27]. However, because waterpipe is a culturally related phenomenon, qualitative studies are needed to understand the relevant factors in each society [28].

In the present study, socio-cultural aspects were classified into two sub-categories: social norm and family norm. Waterpipe in Iranian society opposes gender stereotypes and social stigma and has become popular among women and girls, and young people in recent years [29]. The results of a qualitative study in northern Iran showed that waterpipe in Iran has less stigma than cigarettes. This belief has led to waterpipe being accepted as a traditional and safe pastime in many families and has become a social norm [30]. In some Arab countries, where gender discrimination is a common issue, the stigma of WPS has diminished in recent years, and women are more likely to smoke waterpipe freely and without shame [31].

In this study, consistent with other studies in Iran, participants pointed to the effective role of the family in initiating or continuing WPS [29, 32, 33]. In a qualitative study in Iran, most participants emphasized the role of the family in starting the WPS and believed that the patterns, conditions and educational system of families play a key role in orienting family members towards WPS [34]. In the present study, participants believed that waterpipe use was more prevalent among family members in whom WPS was more common as a family tradition, a finding that was consistent with other studies [30, 35].

Access to waterpipe as a socio-environmental factor has been emphasized in literature related to the prevalence of WPS. From the perspective of the participants in a qualitative study, the supply of waterpipe in public places, especially in cafes and restaurants has helped to increase its use and transfer consumption from homes to the public sphere [36]. Cafes and restaurants provide waterpipe for people who do not have the facilities or patience to prepare it. Waterpipe consumption requires the purchase of the device, tobacco and all its accessories. This is time consuming but all these problems have been solved by cafes and restaurants [36]. Other studies have confirmed that access to waterpipe in traditional cafes increases the chances of WPS among young people [35, 37].

The availability of waterpipe in the social environment and its comorbidity with the lack of facilities for spending leisure time in the community is another important environmental factor that has led young people to use waterpipe. The results of a study in Iran showed that the most important factor affecting WPS from the students’ point of view was spending leisure time [38]. It seems that one of the most important ways to reduce WPS in society is implementing policies to reduce its access and supply in public [26, 30].

Unemployment was another socio-environmental factor of WPS that was expressed by some participants in the present study. They believed that unemployment and consequently having free time was one of the factors that started WPS or increased their consumption. Other studies have shown that the prevalence of WPS among unemployed people was higher than other occupational groups [33, 39].

In this study, social relations was another factor related to WPS, which refers to the impact of peers and friends on waterpipe use in young people. The results of a study showed that the number of days that adolescents and young people spend with friends is directly related to their chances of using waterpipe [40]. The results of another study showed that peer pressure more than curiosity led adolescents to use waterpipe [23]. It seems that the main mechanism of peer influence on WPS is learning how to use waterpipe in friendly or two-person groups [14, 41, 42].

### Individual aspects of waterpipe use

In this study, we found that individual aspects, especially psychological, are related to hookah use in young people. The sub-category of “motivational aspects” included two semantic codes: “appeal” and “relaxing”. Many participants emphasized that waterpipe as a means of smoking has special charms and fundamental differences with cigarettes, which has increased its popularity. In a qualitative study, waterpipe users believed that the appeal of WPS was multifaceted. Taste, smell, and sight were significant sensory cues that contributed to its attractiveness. The use of a wide range of flavoring tobaccos, and innovations associated with large volumes of smoke also contribute to the attractiveness of waterpipe [43]. A qualitative study in Saudi women who used waterpipe showed that the smell and taste of tobacco in different flavors such as cappuccino, chewing gum, and various fruits played an important role in the attractiveness of waterpipe [31].

Another motivating factor for WPS expressed by the participants in this study was the relaxing effect of waterpipe. Similarly, Saffarzadegan et al., in their study concluded that the relaxation seeking is one of the main factors in the tendency of young people to waterpipe [32]. In a qualitative study, Iranian Turkmen men did not mention the relaxing effect of WPS [44]. Other studies have shown that positive perceptions related to waterpipe such as relaxation, encourage and maintain WPS [45, 46]. Given the findings that emphasize relaxation as a key feature of WPS, interventions should focus on providing alternative methods that can meet this need in young people instead of waterpipe.

In the present study, lack of two psycho-protective aspects against waterpipe was identified, which included risk perception and self-efficacy. Consistent with many other quantitative and qualitative studies in the world, the results of the present study showed that the perceived risk related to the health effects of waterpipe and its addictive nature was low in consumers. Participants in the study believed that waterpipe was less dangerous and addictive than smoking. In a similar study, those who smoked cigarette and waterpipe reported that waterpipe is less dangerous, less addictive than cigarettes, and “lighter”, “cooler” and “milder” than cigarette smoke [43]. In other studies in Lebanon and the United States, young people have also emphasized that waterpipe is less dangerous than cigarettes [47, 48]. Participants in this study, in line with the study of Jawad et al. [43], reported that harmful chemicals are filtered due to the passage of smoke through the water in waterpipe, and this process reduces its risks and harms compared to cigarettes. Therefore, it is necessary to implement educational interventions to increase people’s knowledge about the amount of nicotine in waterpipe tobacco, the possibility of addiction, and the inefficiency of passing smoke through water in reducing nicotine.

Self-efficacy is one of the most important personal factors that is used both to resist the temptation to smoke in public and to stop waterpipe among smokers [49]. In WPS behavior, there is an interaction between behavioral, personal and environmental factors, and the process of interactions may affect a teenager’s belief in the temptation to smoke a waterpipe. The results of a cross-sectional study in Iranian male adolescents showed that lack of self-efficacy was the strongest determinant of WPS [50] which was consistent with similar studies [51, 52]. Another study also found that most women who use waterpipe cited poor self-efficacy as an important factor in WPS. They also stated that they could not resist the temptation to waterpipe use in difficult conditions [34].

One of the most important limitations of this study was the uncertainty or unwelcome attitude of some young people, which led to a longer data collection time. Also, in some cases, the owners of the cafeterias prevented the interview in their cafeteria. Moreover, the effect of ethnicity can be considered as a limitation for the generalizability of the results of this study.

## Conclusions

The results of the present study showed that social environment, culture and relations were social aspects of WPS in youths. It seems that the implementation of the policy of reducing access to waterpipe in public environments can reduce WPS. Individual aspects such as motivation, beliefs and psycho-protective also were related to the WPS in participants. It is suggested that educational and interventions, based on models and theories such as health belief model (HBM), theory of planned behavior (TPB), and extended parallel process model (EPPM) be implemented in order to increase young people’s belief and perception on dangers of WPS, and to improve their self-efficacy to smoking cessation.

## Data Availability

The data sets used and analyzed in this study are available from the corresponding author on reasonable request.

## Abbreviations

EMR: Eastern Mediterranean region
NCDs: noncommunicable diseases
WPS: waterpipe smoking
